# Scalable Superabsorbers and Color Filters Based on Earth-Abundant Materials

**DOI:** 10.1021/acsaom.2c00159

**Published:** 2023-01-31

**Authors:** Tao Gong, Peifen Lyu, Marina S. Leite

**Affiliations:** § Department of Materials Science and Engineering, University of California, Davis, Davis, California 95616, United States; ¶ Department of Electrical and Computer Engineering, University of California, Davis, Davis, California 95616, United States

**Keywords:** superabsorbers, color filters, magnesium, earth-abundant, CMOS-compatible, ultrathin film, color transformation

## Abstract

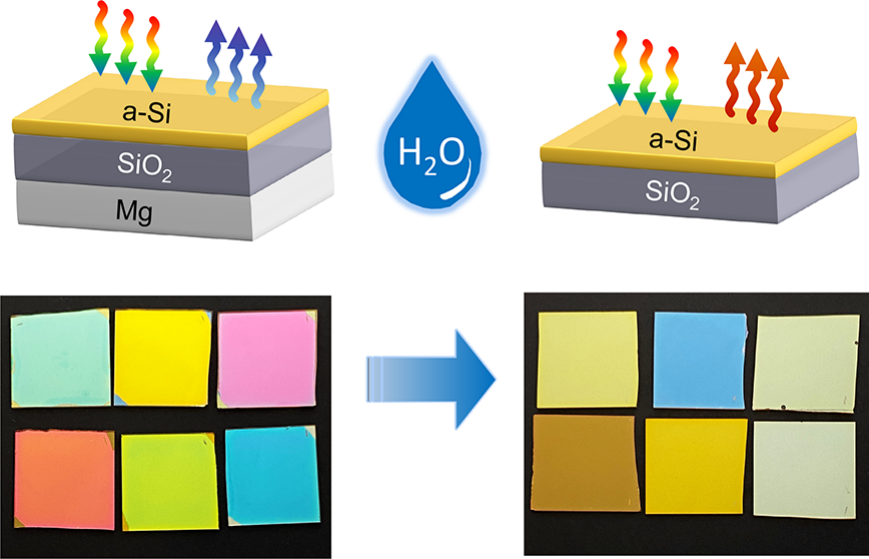

Optical materials based on unconventional plasmonic metals (e.g., magnesium) have lately driven rising research interest for the quest of possibilities in nanophotonic applications. Several favorable attributes of Mg, such as earth abundancy, lightweight, biocompatibility/biodegradability, and its active reactions with water or hydrogen, have underpinned its emergence as an alternative nanophotonic material. Here, we experimentally demonstrate a thin film-based optical device composed exclusively of earth-abundant and complementary metal-oxide semiconductor (CMOS)-compatible materials (i.e., Mg, a-Si, and SiO_2_). The devices can exhibit a spectrally selective and tunable near-unity resonant absorption with an ultrathin a-Si absorbing layer due to the strong interference effect in this high-index and lossy film. Alternatively, they can generate diverse reflective colors by appropriate tuning of the a-Si and SiO_2_ layer thicknesses, including all the primary colors for RGB (red, green, blue) and CMY (cyan, magenta, yellow) color spaces. In addition, the reflective hues of the devices can be notably altered in a zero power-consumption fashion by immersing them in water due to the resulted dissolution of the Mg back-reflection layer. These compelling features in combination with the lithography-free and scalable fabrication steps may promise their adoption in various photonic applications including solar energy harvesting, optical information security, optical modulation, and filtering as well as structure reuse and recycling.

## Introduction

Photonic and optoelectronic devices are omnipresent in technological applications spanning from sustainable energy utilization to advanced computing using photonic/plasmonic integrated circuits (PICs) as well as biosensing and imaging.^1−6^ To date, they often employ conventional metals (e.g., Au, Ag, Cu, Al) and/or their alloys in the key components and building blocks such as in antennas, waveguides, modulators, and detectors.^7,8^ Because the abundant free electrons and low optical loss in these metals can facilitate the excitation of surface plasmon or photonic resonances, these resonances lead to strong optical responses especially in the visible (VIS) and near-infrared (NIR) wavelength regimes. However, these metals are subject to certain limitations such as high-cost (e.g., Au, Ag), complementary metal-oxide semiconductor (CMOS) incompatibility (e.g., Au, Ag), bioincompatibility (e.g., Ag, Cu, Al), and the lack of adaptivity postfabrication, thrusting the continued search for alternative materials.^9−11^

Recently, magnesium (Mg) has emerged as one of such alternative materials used in photonics thanks to its appealing properties.^26^ First of all, Mg can support surface plasmon resonances in a remarkably broad wavelength range covering the entire near-ultraviolet (NUV)-VIS-NIR and with a competitive resonance quality to the noble metals.^12,13^ In fact, the optical loss of Mg is even smaller than Au, Ag, Cu, and Al in the NUV regime, which has positioned Mg on a vantage point for UV applications and sensing.^14,15^ Besides, the hexagonal close-packed (HCP) crystal structure and appropriate twinning of Mg enables the colloidal synthesis of nanoparticles with new sets of shapes that are not available with a face-centered cubic (FCC) crystal structure in the mainstream metals Au, Ag, Cu, and Al.^16^ Further, Mg is low-cost, CMOS compatible, and biodegradable, which facilitate large-scale manufacturing and human health-related usage including drug delivery or cancer treatment.^17^

A characteristic that favorably distinguishes Mg from other mainstream metals is the actively tunable optical properties of Mg via hydrogenation (e.g., hydrogen loading or solid-state proton pumping) and dehydrogenation (e.g., exposure to oxygen), where the state of Mg switches between the metallic (original) and dielectric (hydride) state.^18−21^ Concomitantly, Mg has also manifested transient behaviors when exposed to water or moisture due to irreversible chemical reactions.^22^ Over the years, various Mg-based optical devices with enticing dynamic or transient functionality, including color filtering, holography, and photocatalysis, have been successfully demonstrated.^4,11,23−27^ However, hydrogen-loading typically involves cumbersome setups, while transient behaviors via water exposure have not exhibited superior photon absorption or generated all primary colors for printing and display purposes, impeding their use in many applications.

In this work, we experimentally demonstrate a type of structurally simple superabsorbers in the visible regime constituted by Mg, amorphous silicon (a-Si), and SiO_2_, all of which are earth-abundant and CMOS-compatible materials. The devices present a spectrally selective, near-unity absorption with an ultrathin highly absorbing a-Si layer of deep subwavelength thicknesses, coated on a SiO_2_ spacer layer backed by an optically thick Mg film. The absorption resonance can be readily tuned via the control of the a-Si layer thickness. On the other hand, with different combinations of a-Si and SiO_2_ layer thicknesses, we create diverse reflective colors, including all the primary colors for the RGB (red, green, blue) and CMY (cyan, magenta, yellow) color spaces. Additionally, if immersed in water, the Mg back-reflector of the trilayer structures can be dissolved, which breaks the resonance condition in the a-Si ultrathin cavity and alters the perceived colors. Overall, the ultrathin absorbing layer-induced superabsorption as well as the zero-power-involved hue transformation of our devices, in conjunction with the lithography-free, low-cost, and scalable fabrication steps, may hold great promise for applications such as solar energy harvesting, color filtering, optical information storage, and component reuse/repurposing.

## Results and Discussion

**Figure 1:**
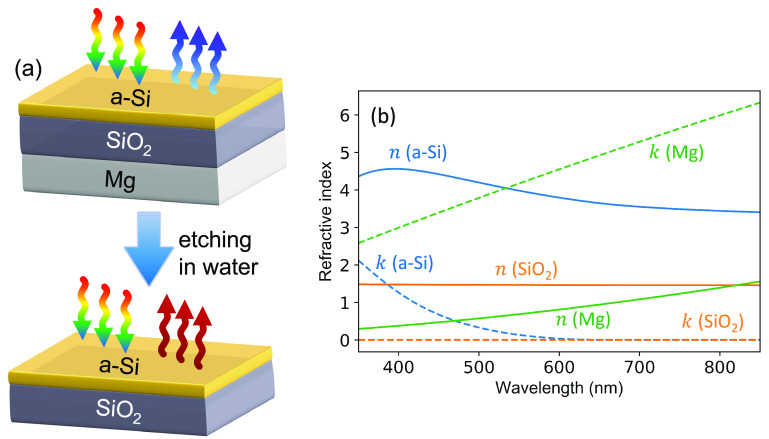
(a) Schematic of the trilayer device consisting of an ultrathin a-Si absorbing layer with varied thickness backed by an SiO_2_-coated optically thick Mg substrate. When immersed in water, the Mg film gradually dissolves, leaving behind a bilayer structure with a different optical response. (b) Measured refractive indices of each material constituting the trilayer stack. Solid lines are the real indices *n* and dashed lines represent the imaginary indices *k* for a-Si (blue), SiO_2_ (orange), and Mg (green), respectively.

We present a photonic device composed of a trilayer stack composed of Mg. The Mg layer acts as a back-reflector and can be removed through etching (dissolution) in water, which would turn the structure into a bilayer stack with a completely different optical response; Figure 1a displays a conceptual representation of this process. The stack is fabricated on a glass substrate where each layer is sequentially deposited: an ultrathin a-Si layer (10–30 nm), a SiO_2_ spacer layer, and an optically thick (160 nm) Mg film; see Figure 1a for the schematic. a-Si and SiO_2_ are well-known and industrially proven materials substantially used in photonic and optoelectronic systems,^28−30^ and they are much more resistant to reaction with water than Mg. The optical properties of these materials (i.e., refractive indices *ñ* = *n* + *ik*) in the visible regime are measured using spectroscopic ellipsometry. As Figure 1b shows, a-Si has a large real index *n* and a high loss *k* in the wavelength range of 400–500 nm, as a result of direct electronic transitions at high photon energies, especially in its amorphous state.^31^

The large indices and high loss of a-Si are key to the operation of our devices. Conceivably, the optical absorption in an ultrathin slab should be weak according to the Beer–Lambert law.^32^ However, the a-Si layer in such trilayer structures can absorb a substantial amount of incident light due to the enhanced light–matter interaction incurred by the strong interference effect in the a-Si nanocavity. Unlike in an archetypal Gires–Tournois etalon composed of a lossless dielectric-coated metal substrate or in a classic asymmetric metal–dielectric–metal (MIM) Fabry–Perot cavity, both of which require the lossless dielectric layer to be at least a quarter-wavelength thick (*d* = λ/4*n*) to satisfy the interference condition,^33^ the resonance in a lossy film can occur with a much smaller thickness. For a cavity formed by a lossy material with comparable values of *n* and *k*, the reflection phase shift at the interfaces of the cavity (i.e., interfaces with air and with the substrate) become significantly different from 0 and π. The propagation phase shift within the cavity can thus be much less than π to realize a total phase difference of π at the top interface, hence drastically diminishing the required cavity thickness.^34,35^

Optical devices built upon ultrathin lossy films have several remarkable advantages. First, the architecture is structurally simple and mechanically stable, and no sophisticated structure design and optimization are involved. Second, a time-consuming and costly nanopatterning process is not necessary so that large-scale manufacturing is viable. Third, the ultrathin absorbing layer can reduce material cost. Fourth, for further development of optical materials for applications in photodetection or photovoltaics, excited charge carriers due to light absorption in the ultrathin semiconductor layer can be efficiently collected at electrodes because of such a small thickness compared to the carrier diffusion length.^36,37^ To date, devices with an ultrathin semiconductor layer (e.g., Si, Ge, and GaAs) on metallic substrate (e.g., Au, Ag, Cu, Al, Cr, and alloys) are attracting increasing attention and have been successfully demonstrated with a wealth of reflective colors/hues produced via various combinations of the materials and thicknesses of the coating layer, owing to the combinatorial effect of the material- and geometry-dependent interface reflection phase shift and propagation phase shift as discussed above.^38−43^

**Figure 2:**
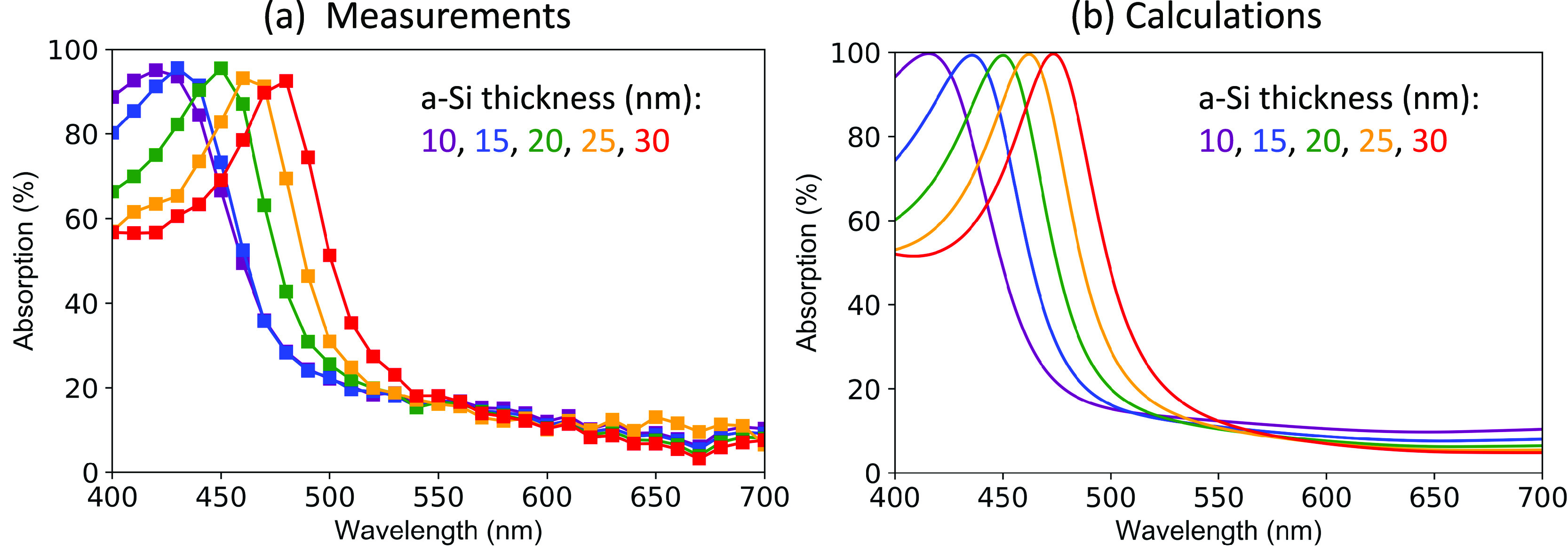
(a) Measured and (b) calculated optical absorption of the trilayer superabsorbers with varying a-Si thicknesses between 10 and 30 nm. The SiO_2_ spacer layer is fixed at 140 nm thickness.

However, in the bilayer structures, the absorbing layer thickness is the only tunable parameter to tailor the optical response, so it is highly dependent upon the intrinsic material properties. To provide a better control over the absorption (reflection), a lossless dielectric spacer layer is sandwiched between the top absorbing ultrathin film and the bottom metal substrate. With the spacer layer, the reflection phase shift at the interface between the ultrathin absorbing layer and the effectively “composite” dielectric-metal substrate can be tuned by adjusting the spacer layer thickness to ensure the optimal interference condition at a given ultrathin layer thickness for an enhanced light–matter interaction. Such phase-compensation strategy has been demonstrated in the optimization of microcavity-enhanced organic photovoltaic (OPV) devices and thin-film optical filters.^44−46^ Our trilayer stacks capitalize on this phase-compensation strategy with the SiO_2_-coated Mg substrate. To achieve the impedance-matching condition for near-unity absorption, the SiO_2_ layer thickness is optimized to be 140 nm for the a-Si thickness of 10–30 nm. Note that, while the SiO_2_ layer plays a critical role in tuning the propagating phase shift, it does not contribute directly to the absorption enhancement as a transparent material; Mg film is still decisive in achieving the perfect absorption. For instance, our calculation shows that the total absorption with only 30 nm a-Si and 140 nm SiO_2_ on a glass substrate is 20.4%, yet the addition of the Mg film boosts the absorption to 99.6%. Figure 2 shows the optical absorption spectra at wavelengths of 400–700 nm measured experimentally and calculated using the transfer-matrix method (TMM) for varying a-Si thicknesses. The calculation results feature a tunable near-unity superabsorption, whereas the measured absorption is slightly lower. This small discrepancy might be attributed to the ∼3.5% reflectivity of incident light upon the glass substrate (assuming a refractive index of ∼1.46 for glass in the visible regime) in combination with the measurement uncertainty. Besides, with a variation of 20 nm in the a-Si layer thickness, the resonant wavelength can be tuned from ∼410 to ∼490 nm, which is in line with the wavelength range where a-Si presents large refractive indices. The resonance tunability is decent considering the ultrathin thickness of the active absorbing layer. Moreover, our calculations show that the major absorption does take place in the a-Si layer (e.g., 83% for 10 nm a-Si) on resonance while the rest is absorbed and dissipated in the Mg substrate due to inevitable field penetration inside the metal.

**Figure 3:**
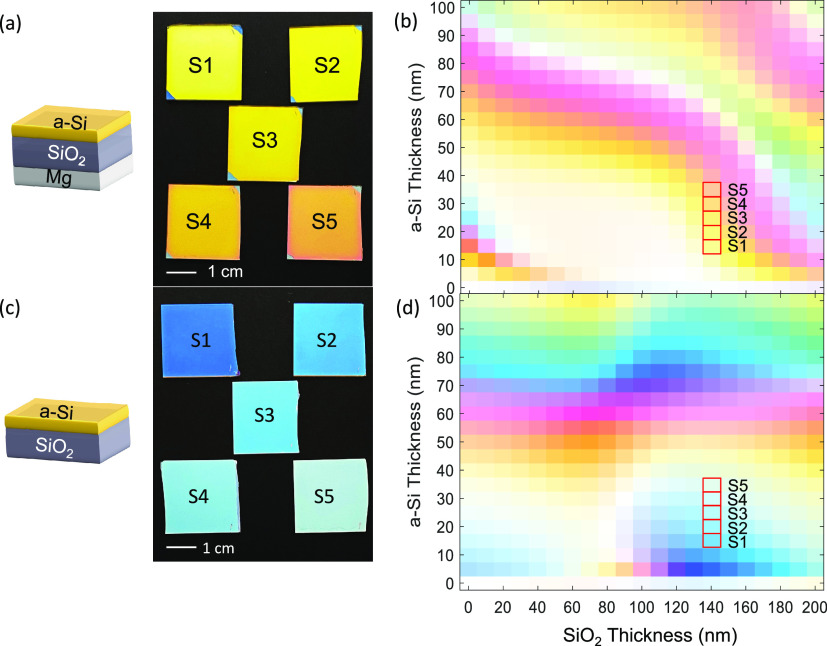
(a) Photograph of the five trilayer superabsorber samples under white light illumination. The SiO_2_ and Mg thicknesses are 140 and 160 nm, respectively, for all samples. The a-Si thicknesses are varied as 10, 15, 20, 25, and 30 nm, corresponding to samples S1, S2, S3, S4, and S5, respectively. (b) Simulated reflective colors of the CIE1931 chromaticity standard for the trilayer superabsorbers with varying a-Si and SiO_2_ thicknesses. Red boxes highlight the thickness combinations corresponding to samples S1–S5. (c) Photograph of the corresponding bilayer samples without the Mg layer but with the same a-Si and SiO_2_ thickness as the superabsorber samples. (d) Simulated reflective colors of the CIE1931 chromaticity standard for the bilayer devices without the Mg layer. Red boxes highlight the thickness combinations corresponding to samples S1–S5.

The near-unity absorption at short wavelengths gives rise to the reflected light mostly distributed at long wavelengths under white light illumination; hence, these superabsorbers yield reflective hues and can also serve as reflective color filters. Figure 3a shows a schematic of the trilayer structures and the photographs of the five different samples taken inside a light box with bright white light illumination, labeled as S1–S5. The five superabsorbers are the same ones whose absorption spectra are measured as shown in Figure 2a with a 140 nm SiO_2_ layer and 160 nm Mg layer and differing a-Si layer thicknesses such as 10, 15, 20, 25, and 30 nm, respectively. All five samples appear yellowish to different extents, which is in qualitative agreement with the absorption spectra in Figure 2a where the near-unity absorption occurs in the blue regime and the fact that blue and yellow are complementary colors according to modern color theory.^47^ To quantitatively depict the reflective colors, we simulate the colors using the CIE chromaticity standard.^48^Figure 3b shows the simulated color palette with different combinations of SiO_2_ and a-Si layer thicknesses for the trilayer structure. The expected colors of samples S1–S5 are highlighted in the palette, matching nicely with the perceived hues of the fabricated devices. In contrast, if the bottom Mg film is absent, the resonance condition in the a-Si nanocavity would break and the near-unity absorption would no longer hold. As such, the reflection of illumination under the same condition would yield different colors. In actuality, the Mg-free bilayer structures corresponding to the above S1–S5 all yield bluish colors to varying extents (see Figure 3c), and the simulated colors also match the perceived hues (Figure 3d). These results indicate that, for the trilayer superabsorbers, removing the Mg back-reflector (e.g., via water immersion) would convert the yellow color pixels into blue ones, which might have technological implications in optical information storage and security where information (e.g., colors in this context) needs to be altered, removed, or rewritten as needed or for novel display and color-filtering applications.

**Figure 4:**
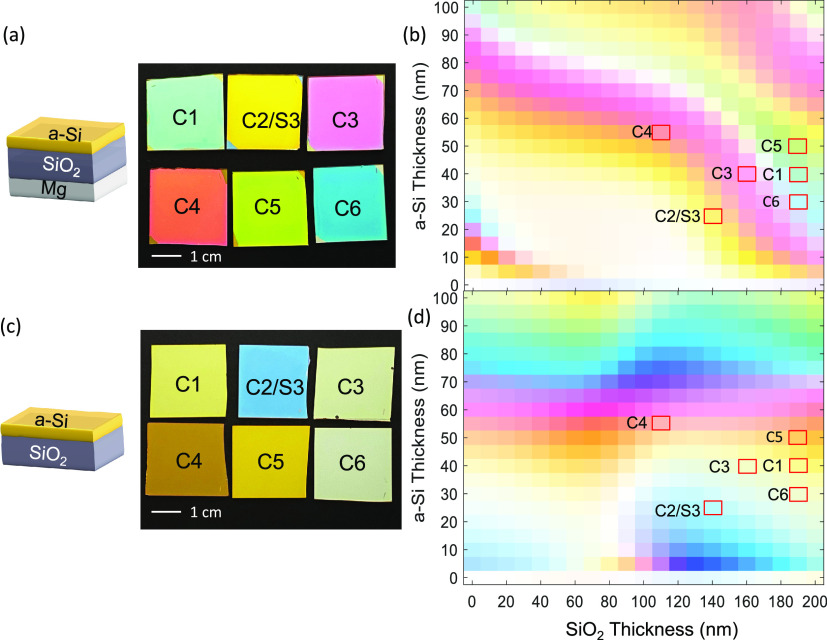
(a) Photograph of the six trilayer color filters under white light illumination. The Mg substrate thickness is 160 nm for all samples. The a-Si layer and SiO_2_ layer thicknesses in the six samples are of various combinations. The upper row shows the cyan, yellow, and magenta colors while the lower row shows the red, green, and blue colors. (b) Simulated reflective colors of the CIE1931 chromaticity standard for the trilayer devices with varying a-Si and SiO_2_ thicknesses (note: the color palette is identical to that in Figure 3b). Red boxes highlight the thickness combinations corresponding to samples C1–C6. (c) Photograph of the corresponding bilayer samples without the Mg substrate layer and with the same a-Si and SiO_2_ thickness as the trilayer samples. (d) Simulated reflective colors of the CIE1931 chromaticity standard for the bilayer devices without the Mg layer (note: the color palette is identical to than in Figure 3d). Red boxes highlight the thickness combinations corresponding to samples C1–C6.

If a-Si and SiO_2_ thicknesses are not at specific combinations, the absorption resonance condition inside the a-Si nanocavity would not hold and neither would the near-unity superabsorption. Yet, the reflective colors would still yield. As shown in the color palette in Figure 3b, more diverse colors can be generated with an arbitrary combination of the two parameters. Figure 4a shows the photograph of six different trilayer samples labeled as C1–C6, under the same white light illumination as described above (Note: C2 and S3 are identical devices). The a-Si and SiO_2_ thicknesses for the six samples are highlighted in the simulated color palette in Figure 4b. The six colors are specifically chosen to be cyan, yellow, magenta, red, green, and blue, corresponding to the primary colors for the CMY and RGB color spaces in modern color theory. The wide tunability and the fine control of the hues demonstrate promising applicability of our devices as color pixels. Furthermore, additional colors can be generated if the bottom Mg layer is removed, as shown in Figure 4c,d. Note that the simulated color palettes in Figure 4b,d are identical with the ones in Figure 3b,d, respectively, which simply serve as a background where the corresponding colors of the actual samples are highlighted by red rectangles.

**Figure 5:**
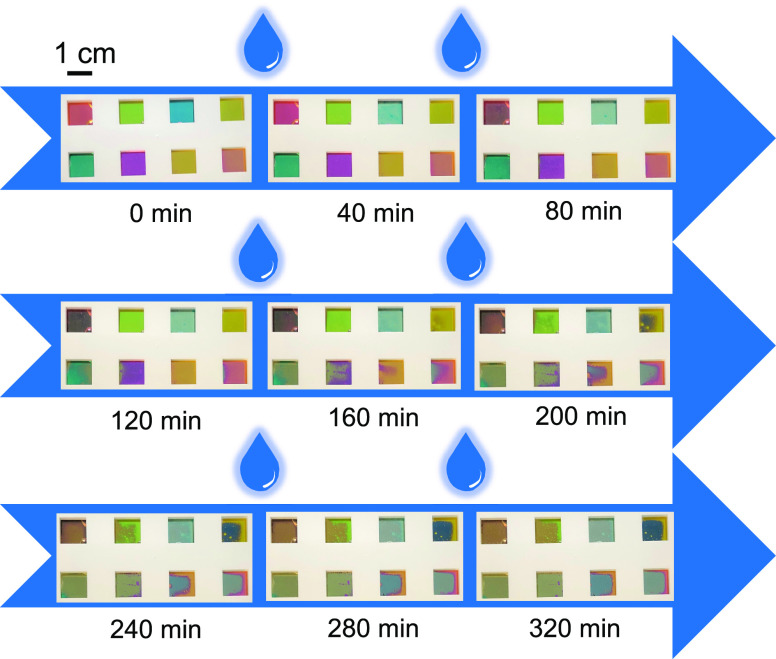
Sequential photographs over time of eight trilayer samples with different reflective hues during immersion in water (without heating and water temperature is 19 °C). The initial colors in the upper row are red (C4), green (C5), blue (C6), and light yellow (S1), and the colors in the lower row are cyan (C1), magenta (C3), yellow (S3), and orange (S5). The initial colors gradually transition into the those for the corresponding bilayer structures as Mg dissolves in water.

The dissolution of Mg in water enables the transformation of hues once the samples are immersed in water. Figure 5 shows such transition process for eight trilayer samples with different initial colors. The eight samples are placed in a mechanical holder with eight squared slots of the size 1 × 1 cm^2^ and then placed in deionized (DI) water (water temperature is measured to be 19 °C in a cleanroom environment). The series of photographs clearly demonstrates the progressive hue transformation of all the devices into their expected final state with only the a-Si and SiO_2_ left after about 320 min. We note here that some experimental artifacts compromise the perceived hues, such as the lighting condition in the cleanroom, photos being taken with samples inside the water, the nonuniform dissolution rate across the samples, and small bubbles and traces arising during the Mg dissolution. Nevertheless, the colors are still in agreement with the expectation, suggesting the practicality of such a hue transformation concept with the structurally simple and possibly reusable color filters. It should be underlined that the etching rate of Mg relies on water temperature. Elevating the water temperature has been found to accelerate the Mg reaction with water.^22^ Indeed, we have performed the test for the same eight samples while the water temperature is held at 40 °C on a hot plate and observed the color transition duration is about half of the time (∼200 min). For applications where the speed of such transition is not critical (e.g., for purposes such as the reuse of substrates), letting the device naturally dissolve at room temperature is sufficient and more importantly completely power-free.

## Conclusions

To summarize, we experimentally realized an all-CMOS-compatible *and* earth-abundant material-based optical device, which can feature spectrally selective near-unity absorption at short wavelengths and can generate diverse reflective hues with appropriate parameter design. Specifically, the superabsorption resonance can be precisely controlled via the tuning of the ultrathin a-Si absorbing layer thickness, while the generated reflective color is the result of a combinatorial tuning of the a-Si and SiO_2_ film thicknesses. Furthermore, the Mg-based constituent allows for a zero-power means of color transformation postfabrication by immersion in water. Our work creates new opportunities for realizing scalable photonic devices for a plethora of applications in energy harvesting, optical information, color display, and component recycling.

## Experimental Details

### Sample Fabrication

All the trilayer devices were fabricated through the sequential depositions of thin films: First, an ultrathin layer of amorphous silicon (a-Si) was deposited on a cleaned glass substrate (1 in. × 1 in.) using the High Density Plasma Chemical Vapor Deposition (HDPCVD) technique (PlasmaTherm Versaline, at the Center for Nano-Micro Manufacturing, CNM2 at UC Davis). The deposition rate was controlled at ∼1.18 nm/s in a vacuum-pumped chamber (5 mTorr chamber pressure) with SiH_4_ and Ar gas flow as 20 and 50 sccm, respectively, and a RF power of 600 W. The root-mean-squared (RMS) roughness of all the a-Si films were found to be 1–2 nm. Such remarkable smoothness can be attributed to the large density of plasmas in the chamber, which planarizes and fills the holes and trenches inside the films through ion bombardment. Second, a SiO_2_ film was deposited in the same HDPCVD chamber at a controlled rate of ∼2.12 nm/s with SiH_4_, O_2_, and Ar gas flow as 28, 56, and 20 sccm, respectively, and RF power of 600 and 100 W, respectively. The subsequent deposition in the same chamber helped to avoid unnecessary contamination on the a-Si surfaces, and the film smoothness can be largely retained. Third, the samples were transferred into a different chamber for electron-beam deposition (AST Peva-600I, at the UC Berkeley Marvell NanoFabrication Lab) of a 2–3 nm MgO capping layer before the final deposition of an optically thick (160 nm) Mg layer (1.5–2.5 Å/s) at a chamber base pressure of 2.5 × 10–3 mTorr.

### Measurement of Materials’ Refractive Indices

The refractive indices of the materials were measured using the Spectroscopic Ellipsometer (J. A. Woollam M-2000, at the Center for Nano-Micro Manufacturing, CNM2 at UC Davis). The reflection spectra at incident angles of 55°, 65°, and 75° and the transmission spectra were collected and then analyzed using the CompleteEASE software by fitting the General Oscillator (Gen-Osc) models for the material’ refractive indices. Each layer was measured separately on the single-layer control samples grown with the same deposition condition and thicknesses on a glass substrate while depositing each layer for the full sample in the same respective chambers.

### Optical Absorption Measurement

The optical absorption spectra were measured by placing the sample inside a 6 in. Labsphere integrating sphere. Chopped illumination from a Fianium@ WhiteLase Supercontinuum laser source was sent into the integrating sphere and impinged onto the sample. Light power bounced off the sample and off the sphere inner walls was collected by a Si photodetector and recorded using a SR830 DSP lock-in amplifier (LIA) for suppressing experimental noises.

### Sample Photographs

The photographs of the samples were acquired using a light box (OrangeMonkie Foldio360 Smart Dome) under white light illumination with full brightness and a color temperature of 5600 K.

### Etching of Samples

All samples were cut into 1 cm × 1 cm pieces and placed inside the squared slots on a mechanical holder. The holder was entirely immersed in DI water in a container as is or placed on a hot plate for heating. A series of photos were taken using smart phone cameras at a specific interval throughout the sample hue transition process.
